# Epidemiology of Hepatitis B Virus (HBV) and Hepatitis C Virus (HCV) Related Hepatocellular Carcinoma

**DOI:** 10.2174/1874357901812010026

**Published:** 2018-02-28

**Authors:** Arnolfo Petruzziello

**Affiliations:** Department of Pathology, Virology and Molecular Biology Unit, Istituto Nazionale Tumori- IRCCS Fondazione G. Pascale, Naples, Italy

**Keywords:** Hepatocellular carcinoma, HBV, HCV, HCC, Epidemiology, Risk factors

## Abstract

**Introduction:**

Hepatocellular carcinoma (HCC) is one of the most prevalent primary malignant tumors and accounts for about 90% of all primary liver cancers. Its distribution varies greatly according to geographic location and it is more common in middle and low- income countries than in developed ones especially in Eastern Asia and Sub Saharan Africa (70% of all new HCCs worldwide), with incidence rates of over 20 per 100,000 individuals.

**Explanation:**

The most important risk factors for HCC are Hepatitis B Virus (HBV) infection, Hepatitis C Virus (HCV) infection, excessive consumption of alcohol and exposition to aflatoxin B1. Its geographic variability and heterogeneity have been widely associated with the different distribution of HBV and HCV infections worldwide.

Chronic HBV infection is one of the leading risk factors for HCC globally accounting for at least 50% cases of primary liver tumors worldwide. Generally, while HBV is the main causative agent in the high incidence HCC areas, HCV is the major etiological factor in low incidence HCC areas, like Western Europe and North America.

**Conclusion:**

HBV-induced HCC is a complex, stepwise process that includes integration of HBV DNA into host DNA at multiple or single sites. On the contrary, the cancerogenesis mechanism of HCV is not completely known and it still remains controversial as to whether HCV itself plays a direct role in the development of tumorigenic progression.

## 1 INTRODUCTION

Hepatocellular carcinoma (HCC) is the most prevalent primary malignant tumor of the liver accounting for about 90% of all primary liver cancers characterized by a high incidence in most populous regions of the world. It is the fifth most common cancer in men worldwide and the seventh among women, with more than 700,000 new cases being diagnosed each year and over 600,000 deaths globally per year, accounting for 9.2% of all new global cancer cases (7.9% in men; 3.7% in women) and the third leading cause of cancer-related death, exceeded only by cancers of stomach and lung [1-3].

Although HCC is not the most frequent cancer, its high mortality related to its low resectability rate, high recurrence after resection and poor response to conservative management, causes a serious worldwide health burden with a mortality rate of 0.95 and 5 years survival of 6.9% [4, 5].

The distribution of HCC varies greatly according to geographic location and it is more common in middle and low- income countries than in developed ones. The disease burden is highest in areas with endemic HBV infection (where HBsAg prevalence is 8% or more), such as in sub-Saharan Africa and especially in Eastern Asia (70% of all new HCCs worldwide), with incidence rates of over 20 per 100,000 individuals. China by itself, for example, with the highest HCC incidence worldwide (395,000 cases per year and an incidence rate of 35 in 100,000), accounts for 55% of liver cancer deaths each year [4, 6, 7]. Mediterranean countries such as Italy, Spain, and Greece and Eastern and Southeastern Europe have intermediate incidence rates of 10-20 per 100,000 individuals, while the Americas and Great Britain have a relatively low incidence (< 5 per 100,000 individuals) [8, 9] despite the incidences having increased appreciably in the USA and some other resource-rich countries during recent years.

The most important risk factors for HCC are Hepatitis B Virus (HBV), Hepatitis C virus (HCV), excessive consumption of alcohol and exposition to aflatoxin B1, but the geographic variability of the incidence of HCC and its heterogeneity has been widely associated to the different distribution of HBV and HCV infections worldwide [10, 11]. Globally HBV accounts for about 80% of virus-associated HCC cases, especially in Africa and East Asia, the highest incidence HCC areas, while HCV infection, involved in about 20% of the total HCC cases, seems to be mainly related to HCC development in low incidence HCC areas like Western Europe and North America [4-8, 12].

Chronic alcohol abuse (> 50-70 g/day for several years) and aflatoxin B1 (AFB1) exposure have been widely described as two of the leading risk factors of HCC. The annual HCC rate among Child Pugh Class A or B alcoholic cirrhosis is about 2.5% and the urinary excretion of aflatoxin metabolites have been described associated with a 4 fold increase in HCC risk [13, 14].

Many studies have tried to identify other risk factors for the so called cryptogenic HCC, liver tumors not directly correlated to the previous risk factors. The rising incidence of HCC especially in the high income countries, like USA, where the occurrence of HCC has tripled during the past 2 decades (from 1.4 per 100,000 in 1975–1977 to 4.8 per 100,000 in 2005–2008) has been attributed mainly to the increasing occurrence of other two risk factors: Non-Alcoholic Fatty Liver Disease (NAFLD) and non-alcoholic steatohepatitis (NASH) [7, 15]. However, it is necessary to state that the association between NASH and HCC is absolutely indirect and related to studies examining HCC risk in presence of obesity and diabetes [16, 17], while NAFLD seems to act synergistically with other risk factors, such as chronic hepatitis C or alcoholic liver damage in HCC progression [18].

Iron overload, either inherited or, in the case of sub-Saharan population, acquired, is a less common risk factors, as Membranous Obstruction of the Inferior Vena Cava (MOIVC) and cigarette smoking. The role of oral contraceptive steroids in tumorigenic progression still remains uncertain.

Concerning the gender susceptibility to HCC, in all geographical regions males have a higher incidence than females, although the ratio is more skewed in regions where the HCC risk is higher. The male: female ratio is approximately 3:1 or 4:1 in the Asia-Pacific region and in sub-Saharan Africa, as well as in medium-risk countries, compared with 2:1 in regions with low HCC incidence [1-5]. The reasons for which males have high risk of developing HCC if compared to females is not completely understood and it may be partially explained considering the male-specific prevalence of specific HCC risk factors, as HBV and/or HCV infections, smoking and alcohol consumption, even if the role of androgenic hormones in predisposing males to the progression to HCC is not clear [19].

The global age distribution of HCC greatly varies. In resource-rich countries (USA, Canada and United Kingdom), HCC is rare in patients under the age of 40 years and its incidence progressively increases in those aged over 65 years, with the highest age specific rate at 75 years [1-7]. Instead, in high risk populations (Asia and sub-Saharan Africa), there is a distinct shift, ranging from Mozambique, where 50% of the HCC patients are less than 30 years of age at the time of diagnosis, and Qidong County, China, where the mean age at the time of diagnosis of HCC is 40 years [20].

The aim of this article is to review key aspects of HCC epidemiology, especially highlighting the role of viral infection in the cancer progression, identifying important areas for future research.

## 2 CHRONIC HBV INFECTION

HBV infection is one of the most prevalent HCC risk factors worldwide and one of the first viruses identified as a cause of a human cancer [21, 22]. In endemic areas, it is often acquired by vertical and perinatal transmission with a chronicity rate > 90%, while in areas of low prevalence such as high income countries, it is usually transmitted horizontally (sexual and parenteral routes) with >90% of acute infections resolving spontaneously.

Chronic HBV infection is one of the leading risk factors for HCC globally accounting for at least 50% cases of primary liver tumors worldwide [23]. Of the approximately 400 million persons worldwide chronically infected with HBV, as many as one-quarter will develop the tumor with an incidence ranging from 340 to 804 per 100,000 HBV-positive males and 120 to 178 per 100,000 positive females per year [23, 24]. It has been widely reported, in fact, that risk of HCC is 15-20 times greater among HBV infected individuals as compared to uninfected population [25, 26] especially in Asia where the incidence rate of HCC was 0.2 per 100 person-years in inactive carriers, 0.6 person-years with chronic HBV infection without cirrhosis, and 3.7 person-years in patients with compensated cirrhosis [27]. In western population the incidence rate seems to be of 0.02 per 100 person-years in inactive carriers, 0.3 in chronic carriers without cirrhosis and 2.2 in subjects with compensated cirrhosis [28].

Although HBV is a notorious HCC cause in the absence of cirrhosis, however, the majority (70%-90%) HBV-related HCC develops in cirrhotic livers [29]. It is well known, in fact, that chronic HBV infection and HCC have a similar geographical distributions, as demonstrated by the fact that HBV chronic infection is implicated in the genesis of as many as 85% of HCC cases in endemic HBV populations, like Chinese and Black African [23, 24, 29].

Several factors seem to increase HCC risk among HBV carriers: demographic (male gender, older age, ethnicity, family history of HCC), viral (high viral load, genotype, longer duration of infection, co-infection with HCV, HIV or HDV), clinical (cirrhosis) and environmental (exposure to aflatoxin, heavy alcohol abuse or cigarette smoking).

It has been widely reported that chronically infected males have a higher risk of developing HCC if compared to females (2:1 to 3:1) (22-24) while in resource-rich countries, HCC is rare in patients under 40 years [30].

In Asian studies, HBV genotype C is associated with more severe liver disease, cirrhosis and the development of HCC, compared with genotype B; whereas in Western Europe and North America, genotype D is more associated with a higher incidence of HCC than genotype A.

Like all forms of carcinogenesis, HBV-induced HCC is a complex, stepwise process that includes viral interaction with endogenous mutagens, such as reactive oxygen species, as well as with the inflammatory process generated by the host’s immune response to the presence of the virus. Anyway, integration of HBV DNA into host DNA at multiple or single sites is thought to be the crucial step in the pathogenesis of HBV-related HCC inducing a series of changes in the surrounding cellular sequences and also perturbing the expression of cellular genes by activating transcription in trans [31]. The HBV x gene, specifically, seems to have a specific role in the carcinogenetic process for its intrinsic property to promote cell cycle progression, inactivation of negative growth regulators, and inhibition of the expression of p53 tumor suppressor gene and other tumor suppressor genes [32].

## 3 CHRONIC HCV INFECTION

HCV infection is well known globally as one of the main risk factor in HCC development even if evidence for its causal role in hepatocarcinogenesis is a little more recent [33, 34]. The rate of HCC progression varies greatly among patients with chronic HCV infection and this is probably due to the existence of a complex interplay between host, viral and environmental factors [35], including older age, male gender, alcohol intake and HCV infection [36-38].

Globally, more than 170 million people today, corresponding to 2.5% of the global population, are chronically infected with HCV [11]. Generally, while HBV is the main causative agent in the high incidence HCC areas, HCV is the major etiological factor of HCC in low incidence HCC areas, like Western Europe and North America [39-42]. In Japan, Italy, and Spain, resource-rich countries with an intermediate incidence of HCC, HCV accounts for as much as 83% of HCCs, and with odds ratios of 40 to 50 [43, 44].

The rate of progression from chronic HCV hepatitis to HCC is variable and numerous factors have been identified as important predictors of progression, some related to the host (older age, longer duration of infection, male sex or alcohol consumption > 50 g/day), others to environment (viral genotype/subtype or viral load) [40-46].

Natural history of HCV infection shows that the progression of chronic HCV hepatitis to cirrhosis is greatly influenced by the age of the patients: 5% of patients under 40 years and 20% of those over 40 years progress to cirrhosis in less than 20 years. HCC risk in chronic HCV patients depends on the severity of fibrosis stage and the rate of progression is approximately 2-6% per year. It has been established that HCV infected patients have a 15-20 fold risk of developing HCC compared with HCV negative patients [13, 47].

While alcohol and other risk factors are proposed to cause HCC because they cause cirrhosis, the direct carcinogenic role of HCV is still controversial, since HCV infection has been also described in HCC patients without cirrhosis [48, 49].

HCV is characterized by a high degree of heterogeneity and classified into seven recognized genotypes on the basis of sequence of the viral genome, each differing at 30%-35% of nucleotide sites [50-52]. The geographic distribution of HCV genotypes is rather complex. The so called “epidemic subtypes” —specifically 1a, 1b, 2a, and 3a— are widely distributed worldwide and account for a great proportion of the totality of HCV cases, especially in high income countries [11, 53]. The so called “endemic” strains, instead, are comparatively more rare and have been restricted for long time in specific regions, as West Africa, Southern Asia, Central Africa and South Eastern Asia [11].

## CONCLUSION

Despite several published studies showing that patients infected with HCV genotype 1b may have a higher risk of developing HCC than those infected with other genotypes [54-57], other authors did not confirm this result [58, 59]. As a consequence, no consensus has yet emerged and the role of HCV genotypes in both accelerating the progression of the disease and as a risk factor for HCC remains to be established.

The cancerogenetic mechanism of HCV is not completely known. Although it is clear that HCV causes hepatic inflammation by complex and not yet well-understood molecular pathways, including direct viral effects and indirect mechanisms involving cytokine pathways, oxidative stress, and steatosis induction [60], it still remains controversial as to whether HCV itself plays a direct role in the development of HCC. Although experimental data suggest that HCV may contribute to HCC by modulating pathways that promote malignant transformation of hepatocytes [61] and HCV core, NS3, and NS5A proteins were shown to be involved in a number of potentially oncogenic pathways in cell culture (resistance to apoptosis and ras oncogene-mediated transformation [62-65] no consensus has emerged yet.

More recently, it has been suggested that HCV core protein mutations (Gln70 and/or Met91) in HCV genotype 1b patients may be closely associated with HCC development and resistance to PEG-IFN/ribavirin (RBV) treatment [66-70].
